# Gene Duplication and Adaptive Evolution of Digestive Proteases in Drosophila arizonae Female Reproductive Tracts

**DOI:** 10.1371/journal.pgen.0030148

**Published:** 2007-08-31

**Authors:** Erin S Kelleher, Willie J Swanson, Therese A Markow

**Affiliations:** 1 Department of Ecology and Evolutionary Biology, University of Arizona, Tucson, Arizona, United States of America; 2 Department of Genome Sciences, University of Washington, Seattle, Washington, United States of America; Fred Hutchinson Cancer Research Center, United States of America

## Abstract

It frequently has been postulated that intersexual coevolution between the male ejaculate and the female reproductive tract is a driving force in the rapid evolution of reproductive proteins. The dearth of research on female tracts, however, presents a major obstacle to empirical tests of this hypothesis. Here, we employ a comparative EST approach to identify 241 candidate female reproductive proteins in *Drosophila arizonae,* a *repleta* group species in which physiological ejaculate–female coevolution has been documented. Thirty-one of these proteins exhibit elevated amino acid substitution rates, making them candidates for molecular coevolution with the male ejaculate. Strikingly, we also discovered 12 unique digestive proteases whose expression is specific to the D. arizonae lower female reproductive tract. These enzymes belong to classes most commonly found in the gastrointestinal tracts of a diverse array of organisms. We show that these proteases are associated with recent, lineage-specific gene duplications in the Drosophila repleta species group, and exhibit strong signatures of positive selection. Observation of adaptive evolution in several female reproductive tract proteins indicates they are active players in the evolution of reproductive tract interactions. Additionally, pervasive gene duplication, adaptive evolution, and rapid acquisition of a novel digestive function by the female reproductive tract points to a novel coevolutionary mechanism of ejaculate–female interaction.

## Author Summary

In a broad range of organisms, including humans, molecular interactions between the male ejaculate and the female reproductive tract play integral roles in sexual reproduction. Although these interactions are essential, the biochemical composition of the male ejaculate can change rapidly over short evolutionary time periods. It is often hypothesized that this rapid evolution reflects a coevolutionary relationship with the female reproductive tract. The paucity of research on females, however, presents a formidable challenge to empirical tests of this hypothesis. In this study, we sought to identify proteins in the female reproductive tracts of D. arizonae that may be interacting or coevolving with the male ejaculate. Unexpectedly, we discovered that D. arizonae females produce an array of “digestive” enzymes in their reproductive tracts. These classes of enzymes are normally found in the gut, where they degrade ingested food for nutritional uptake. In *D. arizonae,* these enzymes have resulted from recent gene duplications, and natural selection has caused rapid and radical changes in their amino acid sequences. We propose that this pattern of duplication and diversification reflects the “female side” of a coevolutionary relationship with the male ejaculate. Exploring the “male side” of this relationship is an important avenue for future research.

## Introduction

Extensive research across a broad range of taxa has revealed that the proteins involved in sexual reproduction often evolve rapidly due to positive selection (reviewed in [1–3]). Although the selective forces that underlie this pattern remain unclear, it frequently has been postulated that adaptive evolution of reproductive proteins may result from intersexual coevolution [1–3]. Indeed, this has been demonstrated in the fertilization proteins of the free-spawning marine gastropod abalone, in which the male protein lysin and its female receptor, vitelline envelope receptor for lysin (VERL), both exhibit signatures of adaptive evolution [4–7]. In internally fertilizing organisms, however, such as mammals or insects, the biochemical interactions between male and female reproductive proteins may be vastly more complex. Reproductive outcomes depend not only on interactions between male and female gamete proteins, but additionally on interactions between male seminal proteins and proteins in the lumen of a female's reproductive tract [8–11].

Fruit flies of the genus *Drosophila* provide an important model system for exploring the function and evolution of reproductive tract interactions (reviewed in 9–12]). In *Drosophila melanogaster,* the male ejaculate comprises just under 100 proteins, several of which are known to stimulate important processes in mated females such as ovulation, oogenesis, and sperm storage (reviewed in [9–11]). Several male proteins either undergo proteolytic cleavage in mated females [13–15], or localize to specific portions of the female reproductive tract [16–18], indicating that ejaculate–female interactions are mediated biochemically by females. Between species, rapid changes in ejaculate composition frequently have resulted in lineage-specific seminal proteins [19–21], many of which may be novel coding sequences [22]. Additionally, molecular evolutionary studies indicate that a significant portion of this ejaculate is subject to positive selection in the *melanogaster* [23–25], *obscura* [26], and *repleta* species groups [27].

By comparison, the female side of reproductive tract interactions has received little attention. Female reproductive tract proteins have been identified transcriptionally only in D. simulans [28], and their functions remain entirely unknown. Furthermore, although several female reproductive tract proteins [28–30] and egg membrane proteins [31] show evidence of positive selection, these analyses largely have been confined to the *melanogaster* species group. It is unclear, therefore, how diversity in female reproductive physiology and mating system across the genus [reviewed in 12,32] is reflected in their reproductive proteins. This overall paucity of research on females presents a major obstacle to understanding the evolution of ejaculate–female interactions and the role of intersexual dynamics in the divergence of reproductive proteins.

Here we use a comparative expressed sequence tag (EST) approach to characterize candidate female reproductive tract proteins in *D. arizonae. D. arizonae* is a *repleta* group species that exhibits important differences from the *melanogaster* group in mating system and female physiology. D. arizonae females remate daily, while D. simulans females wait several days before remating [12]. Female promiscuity may affect the evolution of reproductive proteins by increasing the number of competing male ejaculates [33]. Females of D. arizonae additionally exhibit two remarkable post-mating physiological processes not seen in the *melanogaster* group. First, they incorporate peptide components of the male ejaculate into somatic tissues and oocytes [34], an adaptation which may help defray the cost of egg production during periods of resource limitation [35]. Second, they exhibit an insemination reaction, an opaque white mass of unknown biochemical composition that forms in the female uterus after copulation [36].

By comparing post-mating outcomes in inter- and intrapopulation crosses, several studies have presented evidence for ejaculate–female coevolution in natural populations of D. arizonae and its sister species D. mojavensis (most recent common ancestor, ∼1.5 million years ago [MYA]) [37–41]. Intrapopulation crosses of both species produce larger eggs than interpopulation crosses [38], a process known to be stimulated by several components of the male ejaculate in *D. melanogaster* (reviewed in [9–11]). Additionally, the insemination reaction exhibits a larger size and duration in interpopulation crosses relative to intrapopulation crosses, suggesting this trait is subject to sexually antagonistic coevolution [39]. Finally, desiccation resistance is higher in mated than unmated females [40], and the magnitude of this effect differs between inter- and intrapopulation crosses [41]. Such extensive evidence for physiological coevolution indicates this will be an exciting system to explore the molecular basis of reproductive tract interactions.

Our study identifies 241 candidate female reproductive proteins in *D. arizonae,* of which 31 show elevated rates of amino acid substitution suggestive of adaptive evolution. Unexpectedly, we also discovered three lineage-specific gene families of digestive proteases whose expression is specific to the lower female reproductive tract. These proteins exhibit strong signatures of adaptive evolution, and selected sites cluster near functionally important amino acids. The implications of these findings for ejaculate–female interactions and intersexual coevolution are discussed.

## Results/Discussion

### Functional Classes of Female Reproductive Proteins

**Figure 1:**
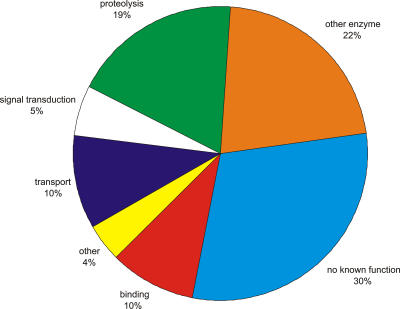
Functional Composition of Candidate Female Reproductive Proteins Functional composition of 241 secreted and transmembrane proteins in D. arizonae female reproductive tracts based on GO terms [59].

We sequenced a total of 2,304 ESTs derived from the *D. arizonae* lower female reproductive tract (parovaria, oviduct, spermathecae, seminal receptacle, and uterus) representing 649 unique proteins (for a complete list see Table S1). Of particular interest are proteins found on cell surfaces or in the lumen of this tissue, which interact directly with the male ejaculate and likely play an integral role in reproductive tract interactions [28]. We therefore designate candidate female reproductive proteins as those that exhibit secreted signal sequences, or transmembrane domains. The gross functional composition of the 241 candidate female reproductive proteins identified in this study (Figure 1) are similar to those of D. simulans [28], and include transport, signal transduction, and proteolysis.

### Rapid Evolution of Female Reproductive Proteins

To explore the evolutionary histories our candidate female reproductive proteins, we calculated the ratio of replacement to silent substitutions (*d_N_/d_S_*) between our D. arizonae ESTs and their orthologs in the D. mojavensis genome. Candidate female reproductive proteins exhibit significantly larger *d_N_/d_S_* values than intracellular proteins in our dataset (median test, *p* > 0.0001), suggesting that these proteins evolve more rapidly than their intracellular counterparts. This elevated rate of amino acid substitution is predicted if adaptive evolution of secreted and transmembrane proteins is a frequent consequence of molecular coevolution with components of the male ejaculate.

Under strict neutrality, only *d_N_/d_S_* ≫ 1 can be considered robust evidence of adaptive evolution. While several of our candidate genes show *d_N_/d_S_* > 1, none of these tests is statistically significant (Table 1). A literature survey has shown, however, that 95% genes that exhibit a pairwise *d_N_/d_S_* > 0.5 contain a class of sites with *d_N_/d_S_* ≫ 1 [28]. Of 227 pairwise comparisons, 31 (14%) were identified with *d_N_/d_S_* > 0.5, indicating they are likely experiencing positive selection (Table 1). This result is largely independent of gene duplication, as the estimated frequency of adaptive evolution it is still 13% when recent duplicates are excluded from the dataset.

**Table 1:**
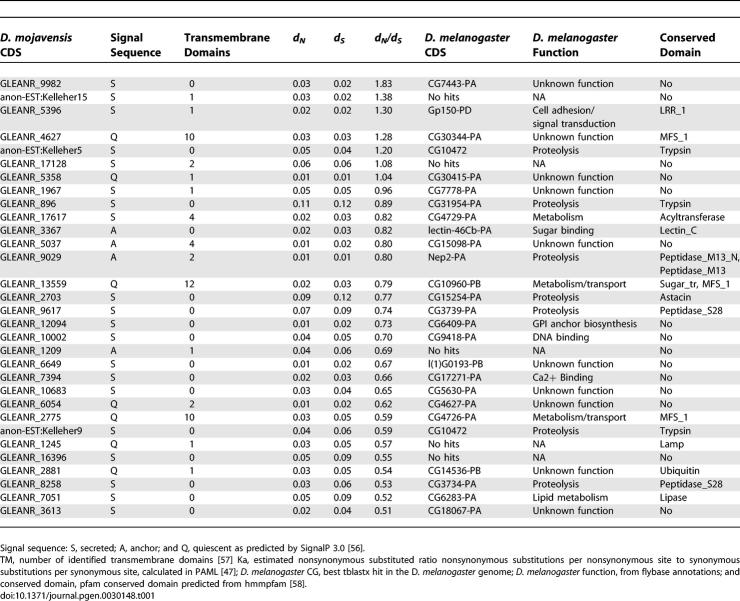
Candidate Female Reproductive Proteins

On a functional level, several protein classes that commonly occur in seminal and fertilization proteins, including lipases, lectins, glycoproteins and proteases, are found in our candidates for adaptive evolution (Table 1). Roughly half of these 31 candidates, however, have no known function, and several others belong to functional classes that are not commonly represented among reproductive proteins. Proteins with unusual or unknown functions make excellent candidates for discovering genes which have acquired novel functions in a biochemical network which likely evolves rapidly. Future studies of these 31 candidates will yield significant insight into the function and evolution of reproductive tract interactions in the *repleta* species group.

### Gene Duplication in Female Reproductive Proteins

Gene duplication plays an integral role in the evolution of D. arizonae female reproductive tract proteins. Specifically, 47% (16) of all secreted proteases in D. arizonae female reproductive tracts have at least one closely related paralog that also is expressed in these same tissues. Duplication events have been extremely recent; as multiple, tandemly-duplicated paralogs in the D. mojavensis genome correspond to only a single gene in D. virilis, the most closely related fully sequenced outgroup (most recent common ancestor, ∼23 MYA; reviewed in [42]). We therefore estimate that the duplication rate of secreted proteases expressed in D. arizonae tracts is 0.0298 (duplications per gene per million years, see Materials and Methods), which is 21-fold higher than the genome wide estimate for D. melanogaster (0.0014, [43]). Although the selective forces involved are yet obscure, such recent and pervasive gene duplication has not been seen in any class of reproductive protein yet studied, including D. simulans female reproductive proteins [28].

**Figure 2:**
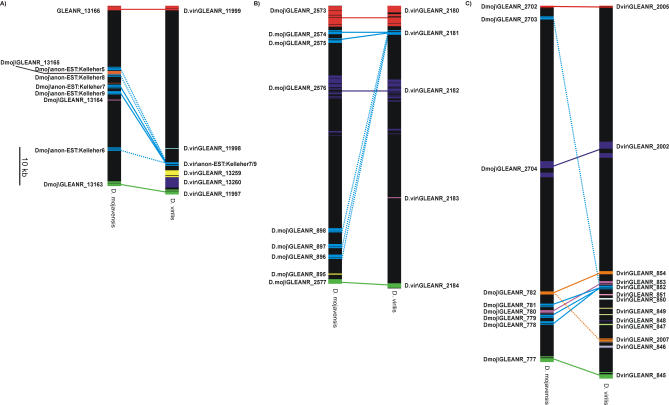
Distribution of Three Protease Gene Families in D. mojavensis and D. virilis Genomes (A) Syntenic regions of protease gene family 1: D. mojavensis Chromosome 4 (scaffold_6680, bp 10216565–10169309) and D. virilis Chromosome 3 (scaffold_13049, bp 10558802–10608251). (B) protease gene family 2: D. mojavensis Chromosome 3 (scaffold_6500, bp 18241557– 18296199) and D. virilis Chromosome 4 (scaffold_12963, bp 15263878–15319561). (C) protease gene family 3: D. mojavensis Chromosome 3 (scaffold_6500, bp 20970182–21063420) and D. virilis chromosome 4 (scaffold_12963 bp 12250368–12347919). Colored blocks indicate individual exons, where each gene is indicated by a different color. Orthologous genes are the same color in both species, and connected by colored lines. Solid lines indicate orthologs with the same orientation, while dotted lines indicate inverted orthologs. Multiple, tandemly duplicated copies in the genome of D. mojavensis correspond to a single gene in the genome of *D. virilis.* Annotation and assembly obtained from unpublished *Drosophila* genomes (http://rana.lbl.gov/drosophila/).

**Figure 3:**
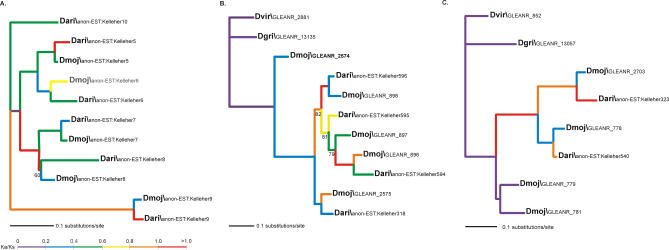
Bayesian Phylogenies of (A) Protease Gene Family 1, (B) Protease Gene Family 2, and (C) Protease Gene Family 3 (A) is midpoint rooted, as D. virilis sequence was too divergent to make an appropriate outgroup. Grey taxon name denotes a pseudogene. Branch colors indicate Ka/Ks values calculated in the codeml package of PAML [47]. Posterior probabilities < 90 are noted.

**Figure 4:**
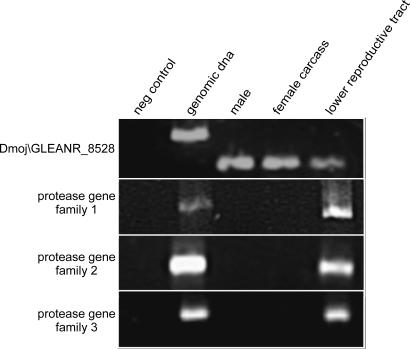
RT-PCR of Three Gene Families Universal primers for each gene family were used to amplify genomic DNA, and cDNA from males, female carcasses (no lower reproductive tract), and lower reproductive tracts (for complete gels see Figure S1).

Four (of 16) duplicated proteases have resulted from two single gene duplication events. The remaining 12 duplicated proteases, however, are associated with small lineage-specific gene families. Each family contains four to six tandemly duplicated paralogs in the genome of D. mojavensis that are syntenic to a single ortholog in the genome of D. virilis (Figure 2). For brevity, we hereafter refer to these three families of tandem duplicates as protease gene family 1, 2, and 3. Phylogenetic analysis of D. arizonae ESTs, and coding sequences from the genomes of *D. mojavensis, D. virilis,* and D. grimshawi (http://rana.lbl.gov/drosophila), reveals the majority of these tandem duplicates in the D. mojavensis genome have a D. arizonae ortholog that is expressed in the lower female reproductive tract (Figure 3). This strongly suggests that the gene duplication events relate in some way to the reproductive function of these proteases. Indeed, reverse transcriptase PCR (RT-PCR) of all three gene families reveals that in adult D. arizonae these genes are exclusively expressed in the lower female reproductive tract (Figure 4). Gene copies present in the D. mojavensis genome that do not correspond to D. arizonae ESTs are likely not highly expressed.

While the function of these duplicated proteins in D. arizonae female reproductive tracts is unknown, they are often similar or identical in their key amino acid residues to several families of digestive proteases found almost exclusively in gastrointestinal tracts (Table 2). Specifically, protease gene families 1 and 2 share appreciable homology with trypsin, chymotrypsin, and elastase, serine endopeptidases commonly found in digestive tracts of both insects and mammals [reviewed in 44]. While, serine endopeptidases can also function in immune signaling cascades across a broad array of organisms, such proteases generally have secondary protein–protein interaction domains that allow for localized regulation of physiological responses [45]. No such domains are seen in either protease gene family 1 or 2, suggesting these proteases exhibit a primarily digestive function. Similar to the two families of serine endopeptidases, protease gene family 3 contains zinc metalloendoproteases very similar to astacin, a prominent digestive enzyme in the crayfish midgut [reviewed in 46]. The reproductive tract-specific expression of these proteases, coupled with recent, lineage-specific gene duplications, suggest that D. arizonae female reproductive tracts recently have acquired a novel digestive function. Digestive enzymes in female reproductive tracts likely have important implications for male reproductive success, and therefore, the evolution of the male ejaculate.

**Table 2:**
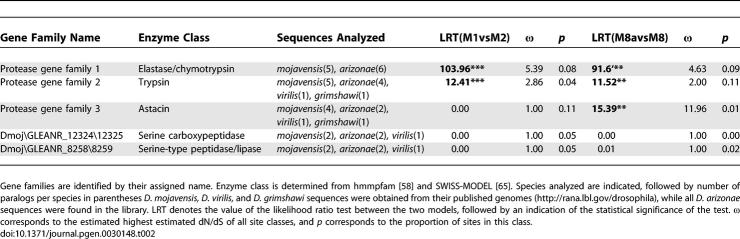
PAML Analysis of Positive Selection in Duplicated Proteases

### Adaptive Evolution of Digestive Proteases

There is compelling evidence that directional selection has played an important role in the evolution of reproductive tract-specific secreted digestive proteases in D. arizonae females. All three families of digestive proteases exhibit a class of sites whose ratio of nonsynonymous to synonymous substitutions (*d_N_/d_S_*) is significantly greater than the neutral expectation of 1 (Table 2). *d_N_/d_S_* values for these selected sites range from 2 to 11.96, indicating certain amino acids in these proteins have experienced strong positive selection. Notably, the two single gene duplication events show no evidence of adaptive evolution (Table 2), indicating that directional selection has been exclusive to the lineage-specific families of digestive proteases.

In order to interpret selection in terms of both duplication and speciation events, we used the PAML free ratios model [47] to estimate *d_N_/d_S_* along every branch in each of the three phylogenies (Figure 3). Positive selection associated with three different speciation events suggests that ongoing changes in the biochemical environment of the female reproductive tract, including possible male contributions to this environment, have resulted in adaptive evolution in some of these proteins. A total of five gene duplication events are also immediately followed by a period of positive selection in one of the paralogous branches (*d_N_/d_S_* > 1), indicating neofunctionalization of a duplicate gene copy. The other seven duplication events however, are followed by elevated amino acid substitution rates (*d_N_/d_S_* = 0.2–1) but no evidence of adaptive evolution. This suggests that relaxed constraint created by functional redundancy between paralogs has also played an important role in the evolution of these gene families.

**Figure 5:**
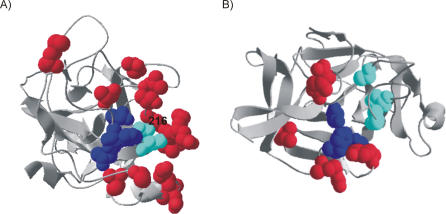
Structural Models Generated in SWISS-MODEL (A) protease gene family 1and (B) protease gene family 2. The blue amino acids comprise the catalytic triad of the active site. The aquamarine amino acids are determinants of substrate specificity [48]. The red amino acids indicate positively selected sites. The labeled amino acid in (A), 216, is a positively selected amino acid that is also a determinant of substrate specificity.

Evidence for adaptive amino acid evolution in duplicated genes implies that selection has acted to diversify the paralogs functionally. Indeed, in all three of the protease gene families, polar, nonpolar, and charged amino acids are seen to inhabit the same selected site in different paralogs. This indicates that directional selection has resulted in recurrent and radical amino acid substitutions, likely affecting the structure and function of the encoded proteins. By mapping selected sites onto predicted molecular structures, it is possible to make more specific inferences about how the biochemical function of these enzymes has been impacted by adaptive evolution. In the two families of serine endopeptidases (protease gene families 1 and 2), positive selection clusters near the catalytic triad: the three amino acids essential for proteolytic function (reviewed in [44]) (Figure 5). Furthermore, in protease gene family 1, positive selection is found adjacent to, and in one case synonymous with, three amino acid sites known to effect substrate specificity (reviewed in [48]). Collectively, these data indicate that directional selection has acted to diversify the catalytic activity of both families of serine endoproteases, and that protease gene family 1 has concomitantly undergone adaptive evolution for increased breadth in substrate specificity. Future functional studies of these enzymes, particularly in terms of how they interact with the male ejaculate, will yield significant insight into the selective pressures that underlie diversification of these extraordinary gene families.

### Implications

Our most striking result was the observation of three lineage-specific radiations of secreted digestive proteases in D. arizonae female reproductive tracts. Although the biological significance of these gene duplications is yet unclear, they may relate to two unusual physiologies exhibited by both D. arizonae and D. mojavensis females. First, the insemination reaction must be degraded by females prior to oviposition or remating [36], a process that could require specialized digestive machinery. Second, female incorporation of ejaculate-derived protein, as observed in D. arizonae and *D. mojavensis,* could be facilitated by degrading seminal proteins and/or sperm into smaller fragments that are more easily absorbed.

Regardless of their physiological function, lower female reproductive–tract specific expression of digestive enzymes points to a novel form of ejaculate–female interaction, in which females may actively degrade, rather than process or activate [13–15], protein components of the male ejaculate. Digestion of seminal proteins or sperm would undoubtedly have important implications for male reproductive success, predicting an evolutionary response from males. Indeed, the association of these proteases with recent gene duplications and strong signatures of adaptive evolution suggests they are involved in an intersexual arms race. Exploring the male side of this interaction, therefore, is an important avenue of future research.

The 31 candidates for adaptive evolution also have important implications for reproductive tract interactions and intersexual coevolution. Roughly half of these proteins have no known function or conserved domain, suggesting they are enriched for novel biochemical functions. Additionally, the candidates include several classes of proteins that have not been implicated previously in reproductive tract interactions. Particularly intriguing are three transmembrane proteins with the conserved transporter domain MFS_1, for inorganic solutes (Table 1). Although the biochemical composition of the *Drosophila* ejaculate is largely unknown outside of its protein constituents, females of several species incorporate ejaculate-derived phosphorus into somatic tissues and oocytes [49]. It is unclear if these transporters underlie such a process in D. arizonae. Their presence and evolutionary history point, however, to nonpeptide biochemical interactions in female reproductive tracts which also may evolve rapidly.

If divergence of reproductive proteins is driven by intersexual dynamics, particularly sexually antagonistic coevolution [50–52], species with more promiscuous mating systems are predicted to exhibit comparatively more adaptive evolution in their reproductive proteins. D. arizonae is significantly more promiscuous than its previously examined congener D. simulans [28], and, consistent with the prediction, we find evidence that this difference in mating system may be reflected in the evolution of their female reproductive proteins. Specifically, we observed that candidate female reproductive proteins in our dataset exhibit higher *d_N_/d_S_* values than intracellular proteins, while this effect was not seen in similar comparisons between D. simulans and D. melanogaster [28]. Additionally, the estimated frequency of adaptive evolution in D. arizonae female reproductive tract proteins (14%) is significantly higher (Fisher's Exact Test *p* = 0.003) than that of D. simulans (5%) [28]. Although the experimental approach for these two studies was quite similar, differences in divergence times between D. arizonae and D. mojavensis (∼1.5 MYA, [37]), and D. simulans and D. melanogaster (∼3 MYA, [53]), could result in more stochastic influence on our measures of *d_N_/d_S_*. Firm conclusions about the effect of mating system on the evolution of female reproductive proteins therefore requires further empirical testing across a broader array of taxa.

Although the function and evolution of male seminal proteins have been researched extensively in both insects and mammals, our understanding of the female reproductive tract proteins with which they interact remains sparse. Our data, as well as previous research in the *melanogaster* group [28–30], indicate that rapid evolution is common among female reproductive tract proteins. We furthermore present compelling evidence that differences in female physiology and possibly mating system between *Drosophila* species are reflected in their reproductive tract proteins. Our research indicates that female reproductive proteins are active players in reproductive tract interactions, and that rapid evolution of seminal proteins must be considered in terms of their relationship with female counterparts.

## Materials and Methods

### Tissue harvesting.

D. arizonae used in this study were collected in December 2005 in Tucson, Arizona by E. S. K. A total of 873 lower reproductive tracts (parovaria, oviduct, spermathecae, seminal receptacle, and uterus) were dissected from mature adult females 9 d or older. In order to maximize transcriptional diversity obtained, dissected females were sampled from a diverse array of mating states. Of the females, 662 were from population bottles, while approximately 40 females were dissected from each of the following treatments: virgin, homospecifically mated 4–8 h postcopulation, homospecifically mated 24 h postcopulation, heterospecifically (to D. mojavensis) mated 4–8 h postcopulation, and heterospecifically mated 24 h postcopulation.

### Library construction.

The harvested tracts were pooled into four separate aliquots of TRIZOL reagent (Invitrogen, http://www.invitrogen.com) and total RNA was extracted according to manufacturer instructions. Quality of these samples was verified with an Agilent 2100 bioanalyzer (http://www.home.agilent.com/), at which point they were pooled. mRNA enrichment was achieved by binding poly-A tails on Oligotex (Qiagen, http://www.qiagen.com/) spin columns. Quality of enriched mRNA was verified with an Agilent 2100 bioanalyzer, and the total yield (1.5 μg) was used for library construction with the Cloneminer cDNA library construction kit (Invitrogen). Approximately 300,000 colony-forming units were obtained with an estimated insert size of 1kb. Of these clones, 10,000 were picked with a QBOT (Genetix, http://www.genetix.com/) operated by the Arizona Genomics Institute (http://www.genome.arizona.edu/). Of these clones, 1,920 were sequenced bidirectionally, and an additional 384 were sequenced exclusively from their 5′ ends. All sequencing was done on at the Arizona Genomics Institute on an ABI 3700 DNA analyzer (https://products.appliedbiosystems.com/) with big-dye terminator chemistry.

### Sequence data analysis.

Base calling and assembly were implemented in Phred and Phrap [54]. All bases with a Phred quality score below 20 (99% accurate) were excluded from further analysis. The estimated frequency of sequencing errors in included bases was 0.04%. BLASTN [55] (*e*-value = 0.01) against the GLEANR coding sequence annotations (from CAF1 assembly http://rana.lbl.gov/drosophila/) of the D. mojavensis genome was used to identify orthologs of D. arizonae ESTs. For ESTs with no good BLASTN hit to annotated coding sequence, BLASTN (*e*-value = 0.01) was implemented against the complete CAF1 assembly of the D. mojavensis genome. ESTs with BLAST hits in the D. mojavensis genome that contained long open reading frames were used to annotate additional genes in D. mojavensis by eye. No examples of ESTs with long open reading frames but no good BLASTN hit in the D. mojavensis genome were identified.

Translations of these coding sequences were used to identify secreted proteins and cell surface receptors using SignalP [56], and transmembrane proteins using TMHMM [57]. Conserved protein family (Pfam) domains were identified with hmmpfam [58]. Gene Ontology (GO) terms [59] were obtained from FlyBase (http://flybase.bio.indiana.edu/) for D. melanogaster homologs, or based on conserved Pfam domains if no D. melanogaster homolog was found. For explicit definitions of GO terms see http://www.geneontology.org/.

In total, the D. arizonae ESTs corresponded to 649 unique proteins in the D. mojavensis genome. The orthologous genes were aligned using CLUSTALW [60] and alignment accuracy was verified by eye. Maximum-likelihood estimates of nonsynonymous substitutions rate (*d_N_*), synonymous substitution rate (*d_S_*), and the ratio of nonsynonymous substitutions per nonsynonymous site to synonymous substitutions per synonymous site *(d_N_ / d_S_)*, were obtained from PAML [47]. For duplicated genes, only reciprocally monophyletic homologs were compared in pairwise analyses.

### Sequence analysis of multigene families.

Sequence data for D. arizonae was obtained from the EST library, while sequences from *D. mojavensis, D. virilis,* and D. grimshawi were obtained from their unpublished, publicly available genomes (http://rana.lbl.gov/drosophila/). GENECONV was used to test for gene conversion between paralogs, using the method of Sawyer [61]. Phylogenetic reconstruction of multigene families was implemented in Mr. Bayes v3.0b4. Nested maximum-likelihood models of codon evolution were implemented in the codeml program of PAML [47] and compared using likelihood ratio tests. Two tests of positive selection were performed. In the first test, the neutral model (M1) is compared with the selection model, in which a class of sites is permitted to exhibit *d_N_/d_S_* (ω) > 1 (M2). In the second test, a beta distribution of site classes in which the most rapidly evolving is fixed to ω = 1 (M8a) is compared to a similar model in which the most rapidly evolving site class is permitted to exhibit ω > 1 (M8) [62]. Multiple initial values of ω were used to ensure convergence on the likelihood optima. For the second test, critical values of the test statistic are determined from Wong et al [63]. Lineage-specific selection patterns of *d_N_/d_S_* were determined by implementing branch-specific models [64].

### Determination of duplication rate.

A total of 34 secreted proteases were identified in D. arizonae female reproductive tracts. Using BLASTN homology and maximum-likelihood phylogenetic reconstruction implemented in PAUP*, we determined these 34 proteins correspond to 37 orthologs in the genome of D. mojavensis, and 23 orthologs in the genome of D. virilis (http://rana.lbl.gov/drosophila/). Assuming no gene conversion or gene loss, the total copy number of these genes was 23 at the divergence of the D. mojavensis and D. virilis lineages. Duplication rate can therefore be estimated by the following exponential growth equation:

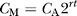



Where *C*_M_ is copy number of D. mojavensis (37), *C*_A_ is the ancestral copy number (23), *t* is the divergence time between D. mojavensis and D. virilis (*t* = 23 MYA [42]), and *r* is the estimated rate of duplication per gene per million years.

### RT-PCR.

D. arizonae RNA was extracted from 20 whole males, 70 reproductively mature females from population bottles lacking their lower reproductive tracts, and 70 lower reproductive tracts preserved in TRIZOL (Invitrogen) according to manufacturer instructions. Purified RNA was treated with DNAseI (Gibco, http://www.invitrogen.com/), and reverse transcribed with the iScript cDNA synthesis kit (Bio-Rad, http://www.bio-rad.com/). Resultant cDNA was diluted to 10 ng/μl, and used as a template for standard PCR using universal primers, with D. arizonae genomic DNA as a positive control. Primer sequences are as follows: Dmoj\GLEANR_8528-F, 5′-AAGAAGCGCACCAAGCACTTCATC-3′; Dmoj\GLEANR_8528-R 5′-TCTGTTGTCGATACCCTTGGGCTT-3′; protease gene family 1 -F1 5′-ATGTGGAATCTAAGCCCAGCCAA-3′; protease gene family 1 -F2 5′-RTAGATGGCAGTTGCTYCTYGTG-3′; protease gene family 1 -R1 5′-GATGYGATACCAATCACRGTGCT-3′; protease gene family 1 -R2 5′-ACGATRCCAATCACRGTGCYAGA-3′; protease gene family 2 -F1 5′-CTCAAACCGCARTAGYTRTCCT-3′; protease gene family 2 -F2 5′-CTTCAAGCCGCMGTWGCTGTCCT-3′; protease gene family 2 -R1 5′-CACCRCTGTGYTYCCTRATCCATTC-3′; protease gene family 2 -R2 5′-CACCGCWGTGCTCYYTGATCCATT-3′; protease gene family 3 -F1 5′-TGAAACCGATCCCAGACTTATAGC-3′; protease gene family 3 -F2 5′-ATGAAACCGATCCCGAGTTGATAG-3′; protease gene family 3 -R1 5′-ATCAGCCATGCTCAATTCTTGTCG-3′; and protease gene family 3 -R2 5′-ATCAGCCCAGCTTAATTCTAGTCG-3′.

### Structural modeling.

Three dimensional structure was predicted by SWISS-MODEL [65], and visualized by Deep View. Selected sites were determined from Bayes Emperical Bayes calculation [66] implemented under M8 in PAML [47].

## Supporting Information

Figure S1 RT-PCR of (A) Dmoj\GLEANR_8528, (B) Protease Gene Family 1, (C) Protease Gene Family 2, and (D) Protease Gene Family 3 One-kilobase markers are indicated. L, DNA ladder; N, negative control; M, whole male cDNA; C, female carcass (no lower reproductive tract) cDNA; R, lower female reproductive tract cDNA.
(7.8 MB TIF)

Table S1 D. arizonae Female Reproductive Tract ESTs D. mojavensis CDS: coding sequence from GLEANR annotations (http://rana.lbl.gov/), D. melanogaster homolog identified by BLAST. SignalP: S, secreted; A, anchor; Q, quiescent as predicted by SignalP 3.0 [56]; TMHMM, number of identified transmembrane domains [57]; Ka, estimated nonsynonymous substitutions per nonsynonymous site; Ks, estimated synonymous substitutions per synonymous site; Ka/Ks, estimated ratio of nonsynonymous substitutions per nonsynonymous site to synonymous substitutions per synonymous site; PROT %ID, Protein % identity; CDS %ID, coding sequence % identity calculated in PAML [47]; Conserved domain, pfam conserved domain predicted from hmmpfam [58].
(171 KB XLS)

### Accession Numbers

All sequences for this study are available from the National Institute for Biotechnology Information (NCBI) GenBank (http://www.ncbi.nlm.nih.gov/Entrez/index.html) accession numbers EV41299147751410–EV41383447752253

## Abbreviations

EST: expressed sequence tag
GO: gene ontology
MYA: million years ago
RT-PCR: reverse transcriptase PCR
